# Exploring the mechanism of Jinlida granules against type 2 diabetes mellitus by an integrative pharmacology strategy

**DOI:** 10.1038/s41598-024-61011-8

**Published:** 2024-05-04

**Authors:** Haiyan Gu, Liang Zhong, Yuxin Zhang, Jinghua Sun, Lipeng Liu, Zanchao Liu

**Affiliations:** 1 Department of Hebei Provincial Key Laboratory of Basic Medicine for Diabetes, The Shijiazhuang Second Hospital, Shijiazhuang, 050000 China; 2 Department of Shijiazhuang Technology Innovation Center of Precision Medicine for Diabetes, The Shijiazhuang Second Hospital, Shijiazhuang, 050000 China

**Keywords:** Computational biology and bioinformatics, Endocrinology

## Abstract

Jinlida granule (JLD) is a Traditional Chinese Medicine (TCM) formula used for the treatment of type 2 diabetes mellitus (T2DM). However, the mechanism of JLD treatment for T2DM is not fully revealed. In this study, we explored the mechanism of JLD against T2DM by an integrative pharmacology strategy. Active components and corresponding targets were retrieved from Traditional Chinese Medicine System Pharmacology (TCMSP), SwissADME and Bioinformatics Analysis Tool for Molecular Mechanisms of Traditional Chinese Medicine Database (BATMAN-TCM) database. T2DM-related targets were obtained from Drugbank and Genecards databases. The protein–protein interaction (PPI) network was constructed and analyzed with STRING (Search Toll for the Retrieval of Interacting Genes/proteins) and Cytoscape to get the key targets. Then, Gene Ontology (GO) and Kyoto Encyclopedia of Gene and Genomes (KEGG) enrichment analyses were performed with the Database for Annotation, Visualization and Integrated Discovery (DAVID). Lastly, the binding capacities and reliability between potential active components and the targets were verified with molecular docking and molecular dynamics simulation. In total, 185 active components and 337 targets of JLD were obtained. 317 targets overlapped with T2DM-related targets. RAC-alpha serine/threonine-protein kinase (AKT1), tumor necrosis factor (TNF), interleukin-6 (IL-6), cellular tumor antigen p53 (TP53), prostaglandin G/H synthase 2 (PTGS2), Caspase-3 (CASP3) and signal transducer and activator of transcription 3 (STAT3) were identified as seven key targets by the topological analysis of the PPI network. GO and KEGG enrichment analyses showed that the effects were primarily associated with gene expression, signal transduction, apoptosis and inflammation. The pathways were mainly enriched in PI3K-AKT signaling pathway and AGE-RAGE signaling pathway in diabetic complications. Molecular docking and molecular dynamics simulation verified the good binding affinity between the key components and targets. The predicted results may provide a theoretical basis for drug screening of JLD and a new insight for the therapeutic effect of JLD on T2DM.

## Introduction

Type 2 diabetes mellitus (T2DM) is a systematic metabolic syndrome, characterized by hyperglycemia. It is caused by insulin resistance in insulin-sensitive tissues and impaired insulin secretion of pancreatic β-cell^1^. Chronic hyperglycemia can cause damage and dysfunction of various tissues, leading to blindness, renal failure, cardiovascular disease, peripheral neuropathy, dementia and cancer^2,3^. The International Diabetes Federation estimated that there were about 536.6 million people with diabetes mellitus in 2021, and the number was expected to increase to 783.2 million by 2045 (T2DM accounting for 90%)^4^. The rapid growth of T2DM poses a threat to global health and an alarming burden of economic development^5,6^. Although some drugs have been used for controlling blood glucose, they are single target and have some adverse effects, such as weight gain, hypoglycemia, gastro-intestinal side effects and so on^7^. So it is necessary to identify other effective medicine for the treatment of T2DM.

Traditional Chinese medicine (TCM) has the characteristics of multi-component and multi-target. It has less toxicity and side effects. TCM has been used for the treatment of T2DM for thousands of years^8^. Jinlida granule (JLD) is composed of 17 herbs (Table S1), which are designed based on the theory of spleen deficiency for the therapy of T2DM^9^. It has been used for the treatment of T2DM as a monomedication or as an adjunctive medication. JLD can greatly reduce the level of HbA1c especially in those with HbA1c > 8.5%, significantly alleviate insulin resistance in patients with hyperinsulinemia (insulin levels > 20 mU/L) and improve β-cell function with hypoinsulinemia (insulin levels ≤ 20 mUI/L)^10^. JLD combining with hypoglycemic agent metformin can effectively improve the standard-reaching rate of blood glucose and clinical symptoms such as thirst, fatigue, polyuria, etc^11^. The studies of mechanisms showed JLD can ameliorate insulin resistance by up-regulating the insulin signaling pathway and reducing skeletal muscle lipid content^12,13^. JLD can also protect pancreatic β-cell from palmitic acid induced injury by activating AMP activated protein kinase (AMPK)^14^. However, due to the complicated components of JLD, the exact mechanism of JLD for the treatment of T2DM has not been fully understood.

Network Pharmacology is based on the theory of systems biology, using bioinformatics and network analysis methods to analyze biological systems. It is an emerging inter discipline to study the mechanism of drug action^15^. The research concept of network pharmacology coincides with the holistic principle of TCM. It has been widely used to reveal the complex mechanism of TCM at molecular level^16^. Network pharmacology analyses the contribution of each active component in TCM and facilitate the understanding of potential effects of the entire formula^17,18^. Liu et al., identified four active components and 130 core targets of deer antler for immunomodulatory mechanisms with network pharmacology^19^. Wang et al., built herbs-components-targets and compounds-targets-pathways network and identified 13 core targets and two main signaling pathways for qingfeiyin in treating acute lung injury^20^. Molecular docking is an in silico method used to predict receptor-ligand binding patterns and affinity^21^. Molecular dynamics can simulate the conformational motion of receptor-ligand binding and provide more information about the receptor-ligand binding mechanism^22^.

In this study, we utilized network pharmacology, molecular docking and molecular dynamics simulation to reveal the bioactive components, potential targets and the signaling pathways of JLD for the treatment of T2DM.

## Results

### Active components and related targets of JLD

**Figure 1:**
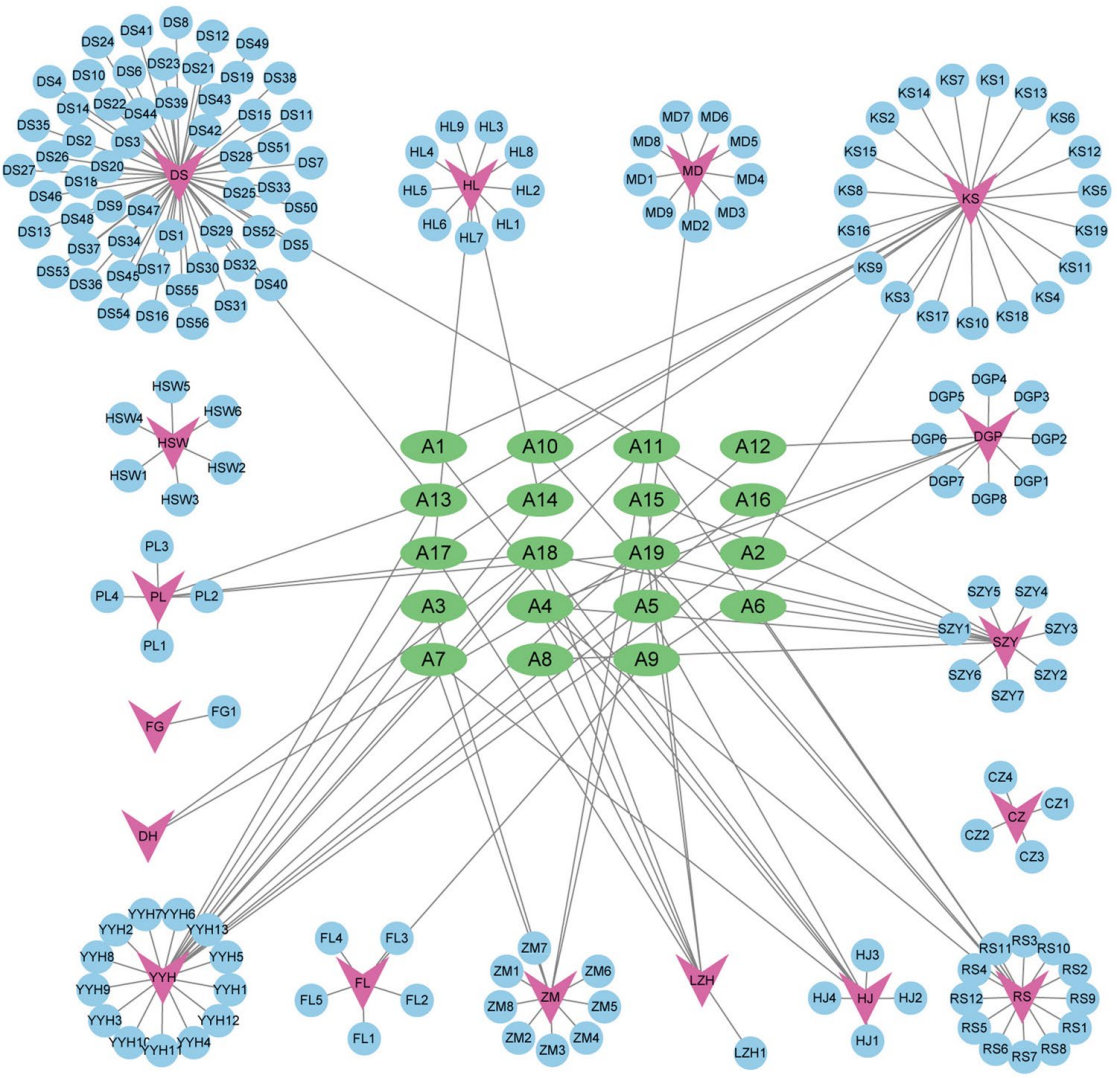
Network of JLD granule-active component. Purple arrows represent herbs in JLD granule. Blue circles represent the related components of each herb. Green ovals are the common components contained in more than one herb. The edges represent the interaction between herbs and active components.

TCMSP, SwissADME, BATMAN-TCM databases and literature were retrieved for active components. The active components without predicted targets were excluded. Finally, 223 components were obtained, 4 active components from Rhizome of Swordlike Atractylodes (Cang Zhu, CZ), 12 from root—bark of Chinese Wolfberry (Di Gu Pi, DGP), 2 from Radix Rehmanniae (Di Huang, DH), 58 from root of Ligulilobe sage (Dan Shen, DS), 6 from Indian Bread (Fu Ling, FL), 9 from Rhizome of Fragrant Solomonseal (Huang Jing, HJ), 11 from rhizome of Chinese Goldthread (Huang Lian, HL), 24 from root of Lightyellow sophora (Ku Shen, KS), 7 from Litchi seed (Li Zhi He, LZH), 7 from Fortune Eupatorium Herb (Pei Lan, PL), 17 from Ginseng (Ren Shen, RS), 14 from Asiatic Cornelian Cherry Fruit (Shan Zhu Yu, SZY), 23 from Epimrdii Herba (Yin Yang Huo, YYH), 12 from rhizome of Common Amarrhe (Zhi Mu, ZM), 1 from Thomson Kudzuvine (Fen Ge, FG) 6 from Tuber Fleeceflower (He Shou Wu, HSW) and 10 from Liriope Equivalent plant: Liriope spicata var prol (Mai Dong, MD). After removing the duplicates, 185 active components were determined. The active components were imported into Cytoscape 3.9.1 to establish the JLD granule-active component network. The analysis results showed that A19 (stigmasterol), A18 (sitosterol), A4 (beta-sitosterol), A13 (luteolin), A17 (quercetin), A11 (kaempferol) and A16 (poriferast-5-en-3beta-ol) were contained in more than 2 herbs (Fig. 1 and Table S2).

The related targets of active components in JLD were first searched in TCMSP database, and then standardize in Uniprot database. Other active components that can not be found in TCMSP database were retrieved in BATMAN-TCM database for their corresponding targets. The targets numbers in CZ, DGP, DH, DS, FL, HJ, HL, KS, LZH, PL, RS, SZY, YYH, ZM, FG, HSW and MD were 58, 80, 29, 133, 22, 83, 169, 189, 166, 75, 109, 65, 208, 105, 3, 74 and 123, respectively. After removing duplicate targets, 337 active components related targets were retained.

### Targets of JLD against T2DM

**Figure 2:**
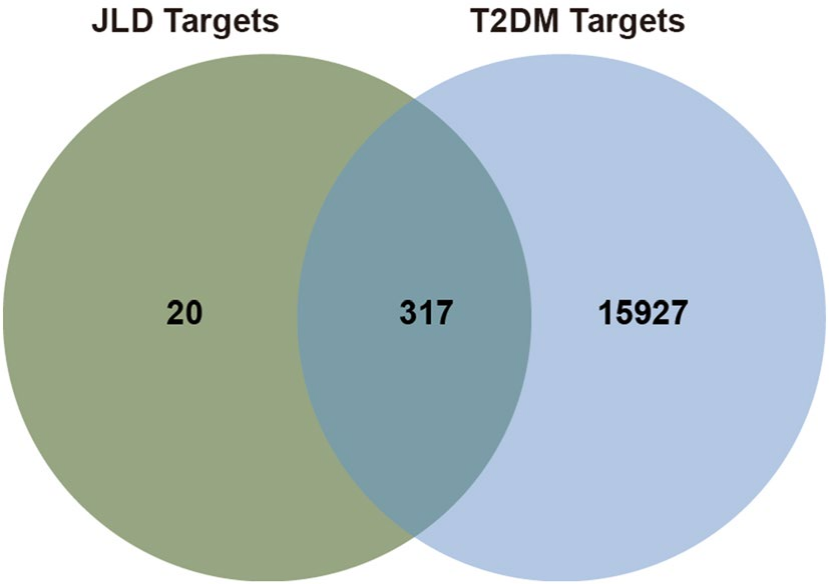
The venn diagram showing the 317 common targets of JLD and T2DM targets.

“Type 2 diabetes”, “Type 2 diabetes mellitus” and “Diabetes, type 2” as the key words were searched in Drugbank and Genecards databases to obtain T2DM related targets. After removing the duplicates, a total of 16,244 targets were obtained. Then the intersection of 337 JLD targets and 16,244 T2DM related targets was analyzed with the Venn diagram. Finally, 317 targets were predicted as JLD targets against T2DM (Fig. 2 and Table S3).

### Active component-disease-target network construction and topological analyses

**Figure 3:**
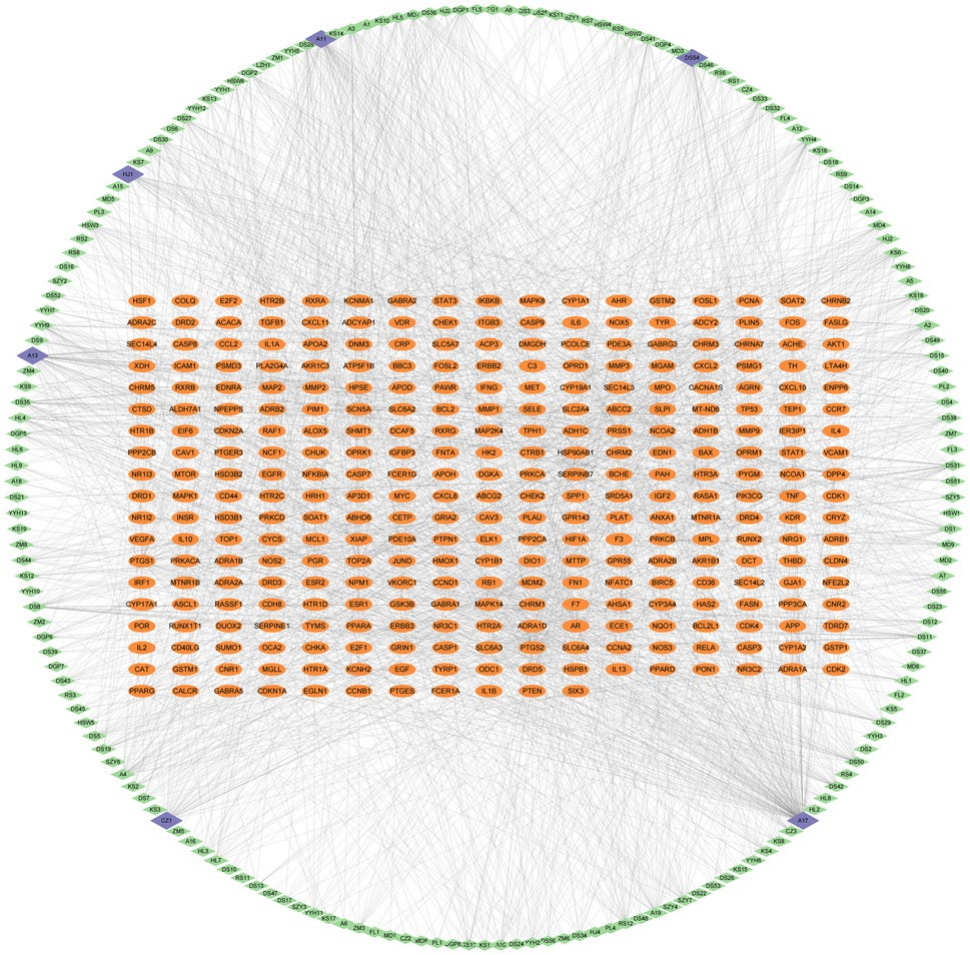
Network of active component-disease-target. Orange ovals represent the 317 common targets of JLD and T2DM targets. Green diamonds represent the disease related active components. Blue diamonds represent the disease related active components with higher degrees. The edges represent the interaction between active components and the common targets of JLD and T2DM targets. The network was constructed by Cytoscape 3.9.1 software.

The active component-disease-target (C-D-T) network was constructed in Cytoscape 3.9.1. The result was shown in Fig. 3. The network contains 502 nodes and 2246 edges. The analyses showed that A17 (quercetin, degree: 136), A11 (kaempferol, degree: 58), A13 (luteolin, degree: 56), CZ1 (wogonin, degree: 44), DS54 (tanshinone iia, degree: 38) and HJ1(baicalein, degree: 34) had the highest degrees. These components may play crucial roles on JLD against T2DM.

### Construction of PPI network and analyses

**Figure 4:**
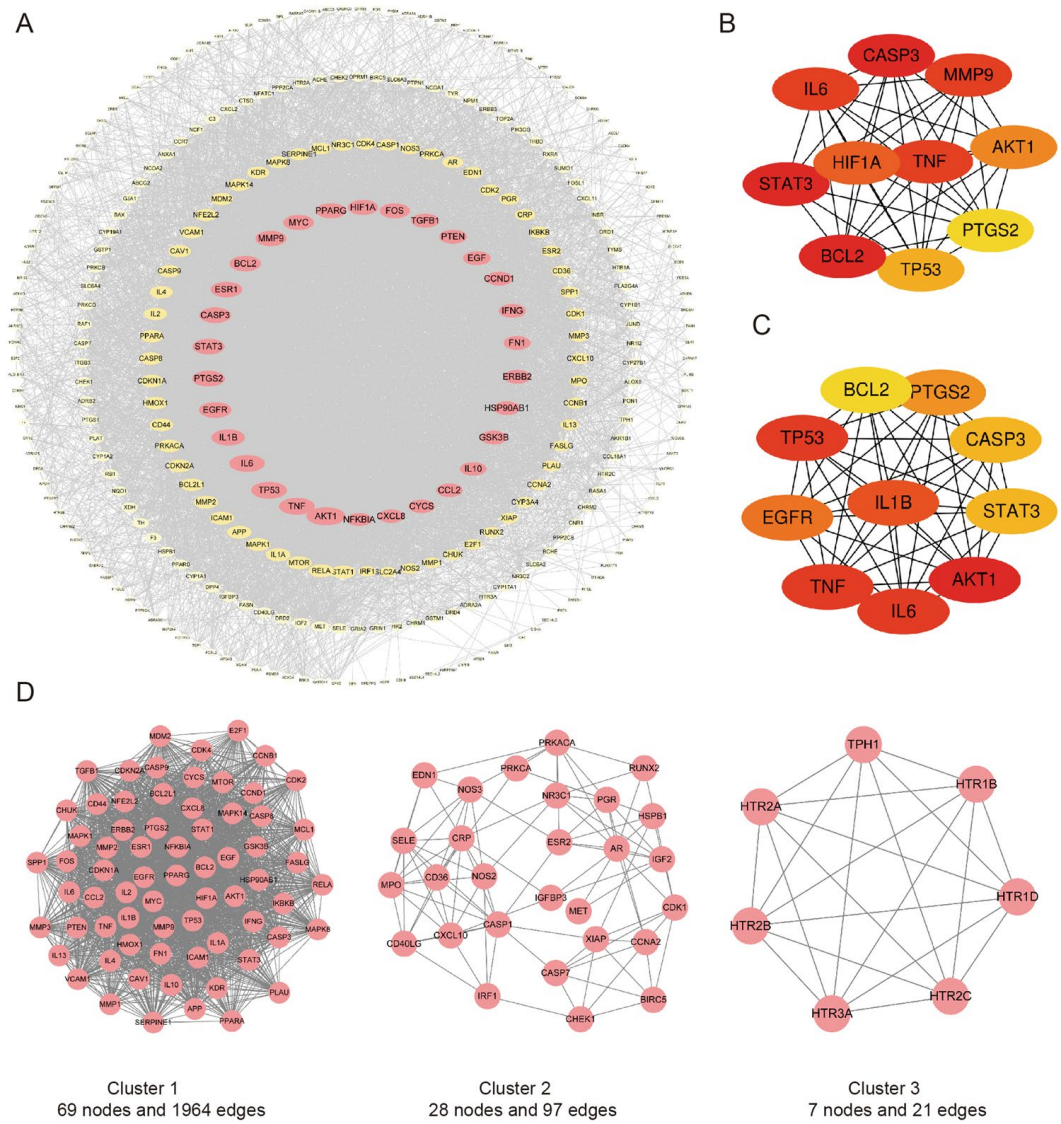
Identification of the key targets by protein–protein interaction (PPI) network analyses. (**A**) The PPI network of therapeutic targets of JLD against T2DM. the ovals represent the therapeutic targets of JLD against T2DM. The size of node is proportional to the target degree centrality (DC) value in the network. The color of node changes from pink to light yellow as the target DC decreases. The top 10 hub targets of the PPI network screened with (**B**) Maximum Neighborhood Component (MNC) and (**C**) Maximal Clique Centrality (MCC). The node color was from red to pale yellow as the corresponding degree decreases. (**D**) PPI network based on cluster analyses by the MCODE plugin.

We submitted the 317 overlapping targets of JLD against T2DM into the STRING database to construct a PPI network. The network contained 317 nodes and 6404 edges. 6 nodes (DCAF5, DIO1, DNM3, IER3IP1, OPRK and TDRD7) were disconnected with interaction score < 0.400. Then, a tsv file about the other 311 targets was downloaded. Finally, the data was imported into Cytoscape 3.9.1 for visualization and analysis. A new network was constructed, which contained 311 nodes and 6404 edges. The CytoNCA plugin in Cytoscape 3.9.1 was used for analysis to get the core targets. RAC-alpha serine/threonine-protein kinase (AKT1), tumor necrosis factor (TNF), interleukin-6 (IL-6), cellular tumor antigen p53 (TP53), interleukin-1 beta (IL1B), prostaglandin G/H synthase 2 (PTGS2), epidermal growth factor receptor 2 (EGFR), Caspase-3 (CASP3), signal transducer and activator of transcription 3 (STAT3) and estrogen receptor (ESR1) were identified as the top 10 targets based on the degree centrality (DC) value, as shown in Fig. 4A and table S4. In CytoHubba module, Maximum Neighborhood Component (MNC) and Maximal Clique Centrality (MCC) were used to get the top 10 hub targets (Fig. 4B, 4C). The intersection of the top 10 core targets with the hub targets may be the key targets of JLD against T2DM. The seven key targets include AKT1, TNF, IL-6, TP53, PTGS2, CASP3 and STAT3. Finally, MCODE was utilized for cluster analyses. The highly connected sub-network with score > 7 were shown in Fig. 4D.

### GO functional and KEGG enrichment analyses

**Figure 5:**
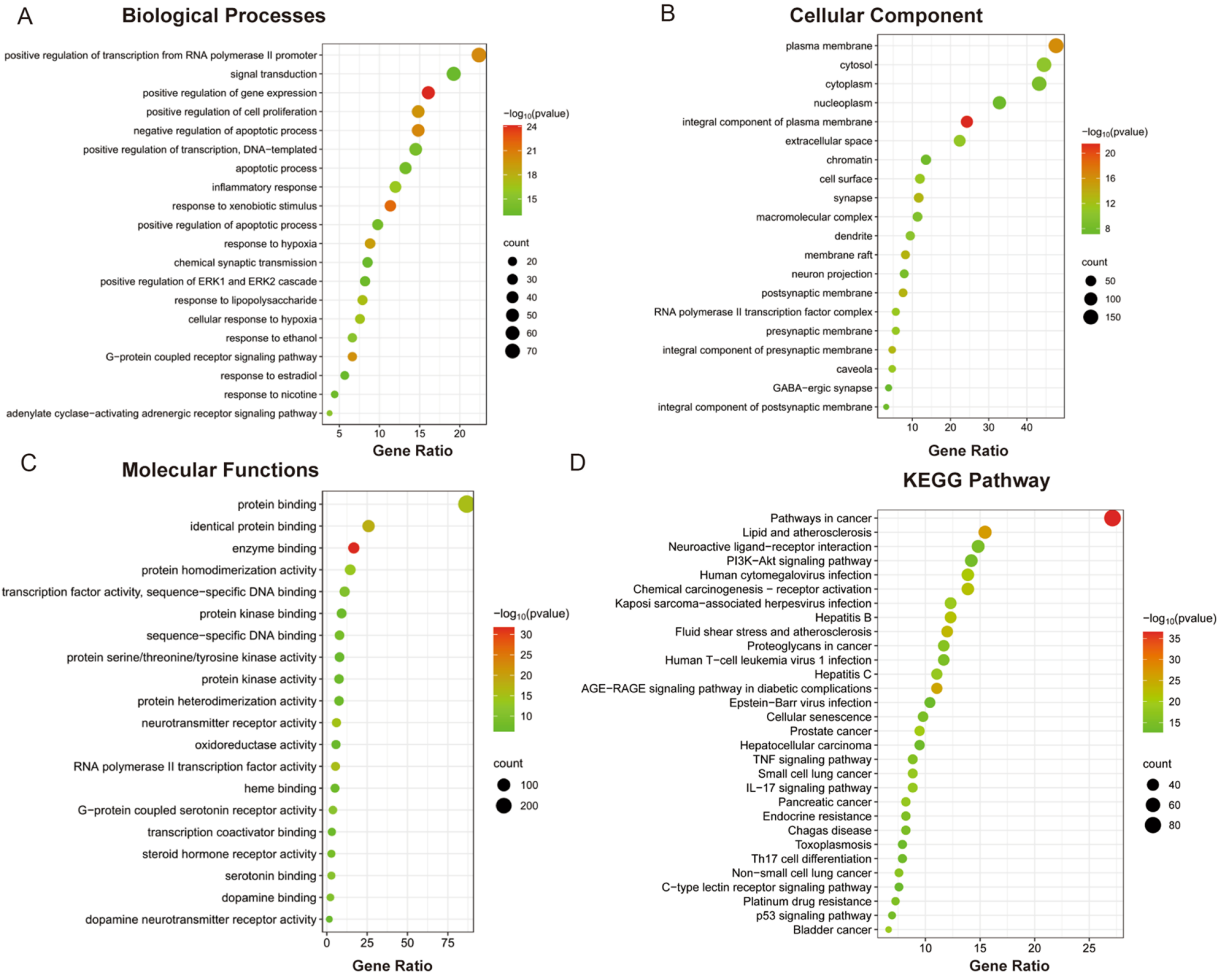
GO (Gene Ontology) and KEGG (Kyoto Encyclopedia of Gene and Genomes) enrichment analyses for 317 overlapping targets. (**A**) Bubble chart showing the top 20 biological processes (BP) terms. (**B**) Bubble chart showing the top 20 cellular component (CC) terms. (**C**) Bubble chart showing the top 20 molecular functions (MF) terms. (**D**) Bubble chart showing the top 30 KEGG pathway. The size of the node is proportional to the gene count enriched in each item. The color of the node changes from red to green as the –log10 (P value) decreased.

317 targets of JLD against T2DM were submitted to DAVID database for GO and KEGG enrichment analysis. Total 1115 GO terms were enriched (*P* < 0.05), including 828 biological processes (BP), 107 cellular components (CC) and 180 molecular functions (MF). The top 20 enriched items with the smallest *P* values in each category were selected and visualized as bubble charts (Fig. 5A–C and Table S5). The *P* value was transferred into –log10 (*P* value) and the color changed from red to green as the –log10 (*P* value) decreased. The size of the node change from large to small with the target count enriched in each term reduced. BP terms were mainly enriched in positive regulation of transcription from RNA polymerase II promoter, signal transduction, positive regulation of gene expression, negative regulation of apoptotic process, positive regulation of cell proliferation, etc. CC terms mainly included plasma membrane, cytosol, cytoplasm, nucleoplasm, integral component of plasma membrane, etc. Highly enriched MF terms were protein binding, identical protein binding, enzyme binding, protein homodimerization activity, transcription factor activity, etc.

178 KEGG pathways were enriched with *P* value less than 0.05. The top 30 significant pathways were presented in Fig. 5D and Table S6. The pathways associated with T2DM mainly include PI3K-AKT signaling pathway, AGE-RAGE signaling pathway in diabetic complications and TNF signaling pathway. It is also involved in other ways, such as lipid and atherosclerosis, fluid shear stress and atherosclerosis, endocrine resistance, cancer related pathways and virus infection pathways. PI3K-AKT signaling pathway (n = 45) and AGE-RAGE signaling pathway in diabetic complications (n = 35) enriched higher number of targets, and may be the important pathways in the treatment of JLD against T2DM.

### Molecular docking validation

**Figure 6:**
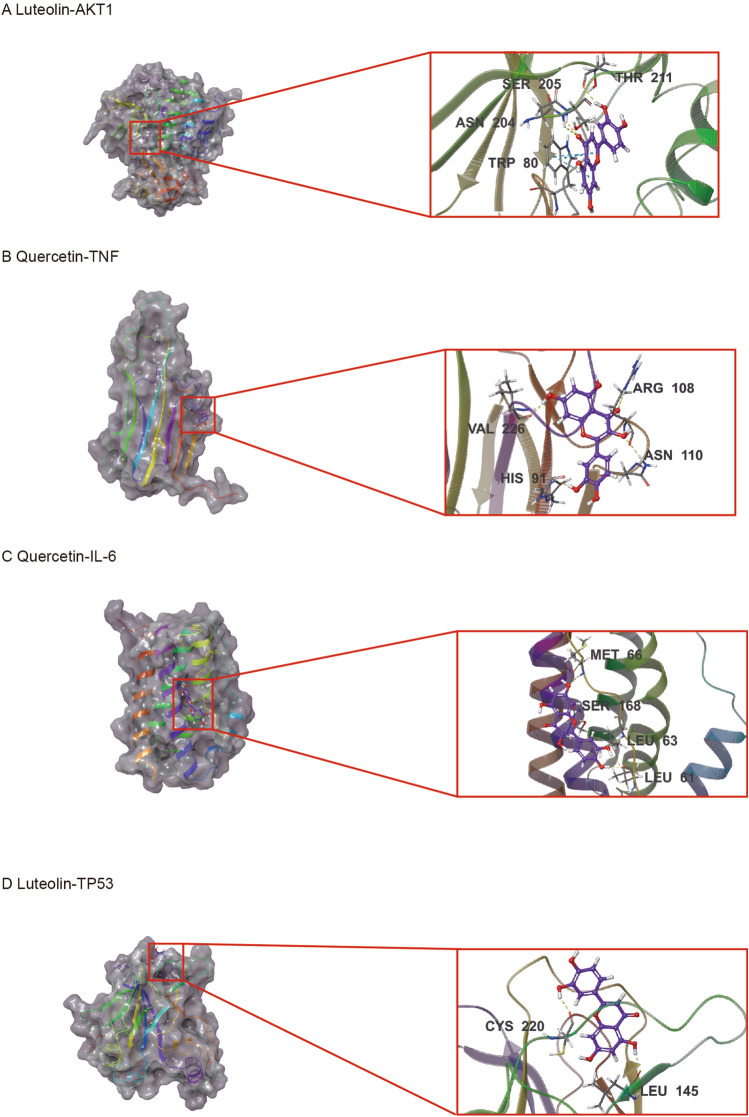

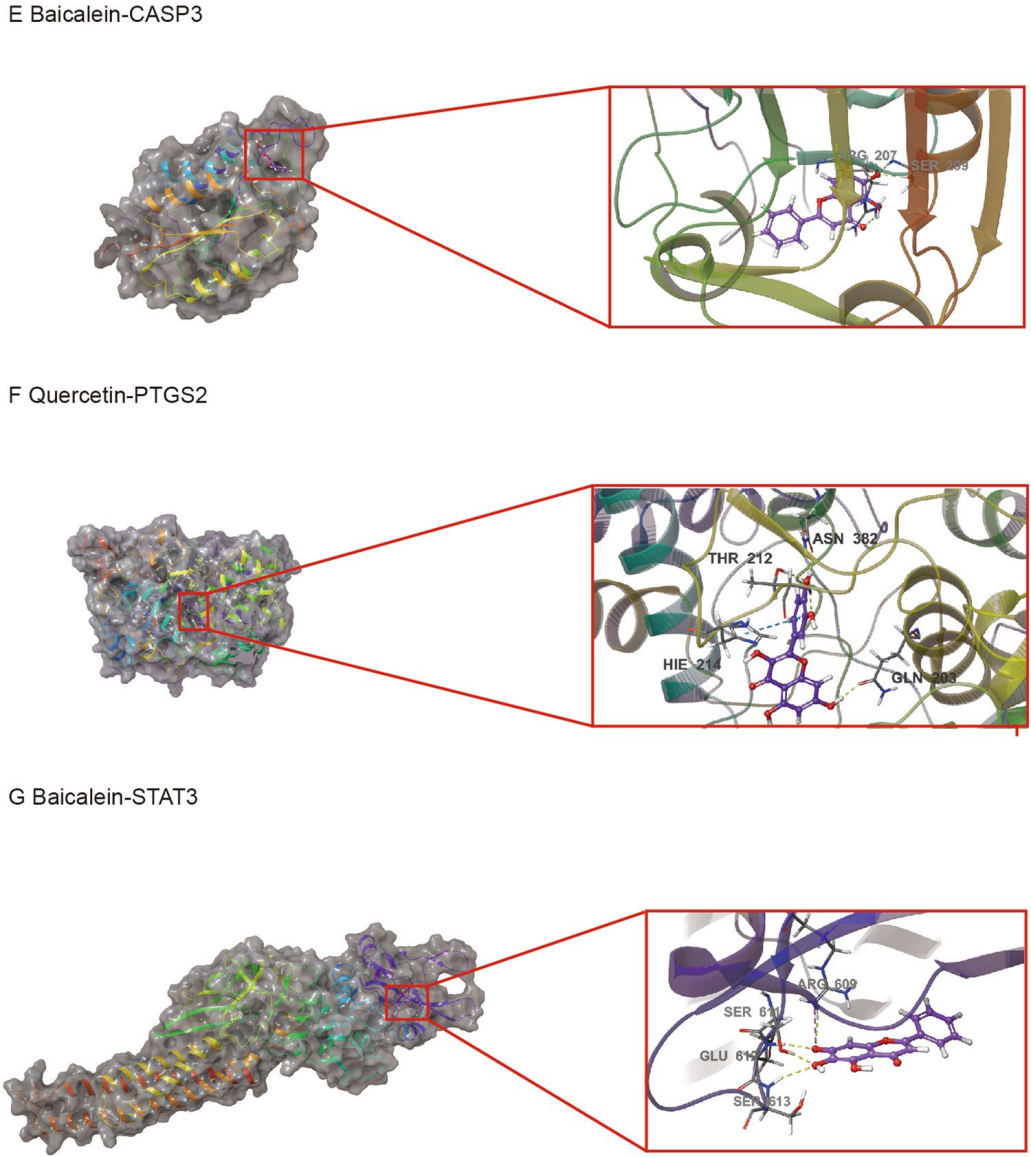
Docking results of the key targets and active components. (**A**) Luteolin-AKT1. (**B**) Quercetin-TNF. (**C**) Quercetin-IL-6. (**D**) Luteolin-TP53. (**E**) Baicalein-CASP3. (**F**) Quercetin-PTGS2. (**G**) Baicalein-STAT3.

According to the PPI network analyses, seven key targets (AKT1, TNF, IL-6, TP53, PTGS2, CASP3 and STAT3) were selected as receptor for molecular docking. 6 components (quercetin, kaempferol, luteolin, wogonin, tanshinone iia and baicalein) with highest degree in the C-D-T network analysis were selected as ligands. The XP Gscore and MM-GBSA dG Bind energies of the key targets and active components are shown in Table 1. XP Gscore less than -6 and GBSA dG Bind less than − 30 kcal/mol indicated high binding affinity. The lower the values, the more stable the binding. AKT1 and TP53 had the best binding performance with luteolin. Luteolin formed one hydrogen bond with AKT1 residues THR211, SER205, and ASN204 respectively and three π-π bonds with TRP80. Luteolin bound with the TP53 by one hydrogen bond residues LEU145 and CYS220 respectively. TNF, IL-6 and PTGS2 had the best docking performance with quercetin. TNF-Quercetin complex had one hydrogen bond with residues VAL226, HIS91, ARG108 and ASN110, respectively. IL-6-Quercetin complex was stabilized by one hydrogen bond with residues SER168, MET66, and LEU63 respectively, and two hydrogen bonds with residue LEU61. PTGS2-Quercetin complex presented one hydrogen bond with residues ASN382 and GLN203, two hydrogen bonds with residue THR212, and a π-π bond with HIE214. STAT3 and CASP3 bound best with baicalein. Baicalein had one hydrogen bond with STAT3 residues SER613, GLU612, and SER611, a hydrogen bond and a salt bridge with residue ARG609. Baicalein formed a hydrogen bond with CASP3 residues ARG207 and SER209, respectively. The details of binding between active sites of these targets and active components are shown in Fig. 6.

**Table 1:** Docking scores of active components with key targets.

Targets	Components	XP Gscore	MM-GBSA dG Bind(kcal/mol)
AKT1	quercetin	− 10.86	− 55.72
	luteolin	− 11.028	− 50.08
	kaempferol	− 9.627	− 44.13
	wogonin	− 9.316	− 50.1
	tanshinone iia	− 8.475	− 47.89
	baicalein	− 4.975	− 25.36
TNF	quercetin	− 7.892	− 37.77
	luteolin	− 6.147	− 36.95
	kaempferol	− 6.048	− 31.17
	wogonin	− 6.287	− 31.65
	tanshinone iia	− 2.198	− 28.56
	baicalein	− 7.878	− 31.1
IL-6	quercetin	− 7.139	− 37.60
	luteolin	− 6.767	− 36.56
	kaempferol	− 5.027	− 37.74
	wogonin	− 3.868	− 26.75
	tanshinone iia	− 2.967	− 26
	baicalein	− 4.718	− 38.29
TP53	quercetin	− 8.428	− 36.37
	luteolin	− 8.905	− 37.65
	kaempferol	− 7.649	− 35.78
	wogonin	− 6.709	− 38.84
	tanshinone iia	− 4.616	− 39.04
	baicalein	− 7.878	− 31.1
CASP3	quercetin	− 6.233	− 41.3
	luteolin	− 5.228	− 42.93
	kaempferol	− 4.512	− 37.82
	wogonin	− 6.114	− 37.87
	tanshinone iia	− 3.262	− 40.52
	baicalein	− 6.431	− 43.76
PTGS2	quercetin	− 6.335	− 31.26
	luteolin	− 6.382	− 20.98
	kaempferol	− 5.634	− 14.59
	wogonin	− 5.698	− 9.95
	tanshinone iia	− 3.57	− 21.12
	baicalein	− 6.697	− 22.38
STAT3	quercetin	− 4.569	− 28.06
	luteolin	− 6.64	− 25.09
	kaempferol	− 5.811	− 24.6
	wogonin	− 3.699	− 28.55
	tanshinone iia	− 2.527	− 36.02
	baicalein	− 6.776	− 45.09

### Molecular dynamics simulation

**Figure 7:**
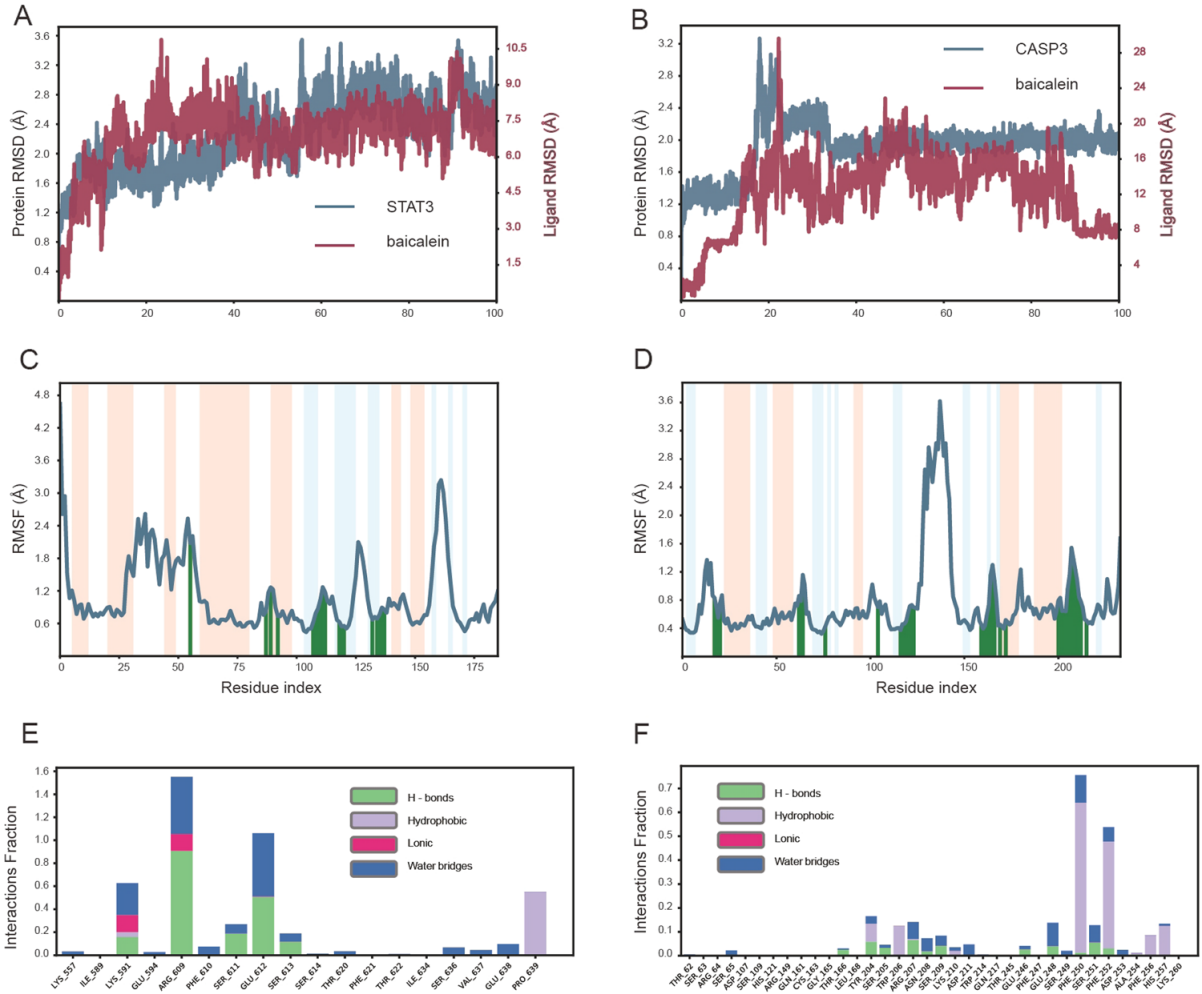
The molecular dynamics simulation of Baicalein-STAT3 and Baicalein-CASP3 complex. (**A**) The root Mean Square Deviation (RMSD) of Baicalein-STAT3 complex. (**B**) The RMSD of Baicalein-CASP3complex. (**C**) The Root Mean Square Fluctuation (RMSF) of Baicalein-STAT3 complex. (**D**) The RMSF of Baicalein-CASP3complex. (**E**) The interaction fraction of Baicalein-STAT3 complex. (**F**) The interaction fraction of Baicalein-CASP3complex.

Molecular dynamics simulation was performed to further verify the results of molecular docking. Based on the molecular docking results, AKT- Luteolin, TNF- Quercetin, IL-6- Quercetin, TP53- Luteolin, PTGS2- Quercetin, STAT3- Baicalein and CASP3- Baicalein had the best binding performance for each key target. The molecular dynamics simulation of AKT1-Luteolin, TNF-Quercetin-, IL-6-Quercetin, TP53-Luteolin and PTGS2-Quercetin have been performed by others^23–26^. In our study, STAT3- Baicalein and CASP3- Baicalein were subjected to molecular dynamics simulation analyses for 100 ns. Root Mean Square Deviation (RMSD) reflects the fluctuation of conformation. A lower fluctuation of RMSD value indicates the conformational stability of simulation process. Figure 7A, 7B showed the RMSD curve of STAT3-Baicalein complex and CASP3-Baicalein complex. STAT3-Baicalein complex was stable after 60 ns. CASP3-Baicalein complex was stable after 40 ns. Root Mean Square Fluctuation (RMSF) can reflect the local fluctuation of protein chain. The peak indicates the larger fluctuation of this region. As shown in Fig. 7C, 7D, STAT3-Baicalein complex have higher flexibility at the residues 25-55aa, 120-130aa and 155-165aa. While CASP3-Baicalein complex have higher flexibility at the residues 120-150aa. As shown in Fig. 7E and 7F, amino acids that played important roles in binding baicalein to STAT3 protein were LYS591, ARG609, GLU612 and PRO639, and their interactions were mainly water bridge and hydrogen bonds. The CASP3 residues of TYR204, PHE250 and PHE252 played important roles in binding to baicalein, mainly by hydrophobic, water bridge and hydrogen bonds .

## Discussion

JLD, a kind of TCM prescription, has been clinically used for the treatment of T2DM in China. Its curative effect as a single or add-on medication has been verified^10,11^. However, the bioactive components and the underlying mechanism of JLD against T2DM remain unclear. In this study, we used network pharmacology, molecular docking and molecular dynamics simulation to explore the potential mechanism of JLD against T2DM.

The active component-disease-target network analysis showed that quercetin, kaempferol, luteolin, wogonin, tanshinone iia and baicalein were associated with more T2DM related targets. These six active components may have important roles on the effect of JLD against T2DM. The efficacies of the six components against T2DM have also been reported previously. Quercetin has the antidiabetic effect by promoting glucose uptake in muscle and liver, reducing the glucose absorption in small intestine, increasing insulin secretion and alleviating ferroptosis of pancreatic β cells^27,28^. Kaempferol can activate AKT to promote glucose metabolism, and stimulate autophagy to protect pancreatic β cells from apoptosis^29^. Luteolin ameliorates hyperglycemia and improve β cells dysfunction by modulating PPAR-γ, SREBP-1c and inflammatory mediators^30^. Wogonin can ameliorate hyperglycemia by activating PPAR-α^31^. Tanshinone iia can alleviate T2DM by inhibiting the expression of inflammation factors^32^. Baicalein can improve glucose metabolism by promoting glucose uptake and glycolysis and inhibiting gluconeogenesis in liver cells^33^.

To identify the key targets of JLD treatment for T2DM, we utilized the 317 overlapping targets of JLD against T2DM to construct a PPI network. By bioinformatics analyses, 7 key targets of JLD treatment for T2DM (AKT1, TNF, IL-6, TP53, PTGS2, CASP3 and STAT3) were obtained. These targets were mainly involved in metabolism, inflammation and apoptosis. AKT1 is an important mediator of glucose metabolism, lipid metabolism, protein synthesis, cell survival and inflammation^34,35^. It will be discussed in more detail as follows. TP53 is associated with metabolism^36^. Study has reported that the TP53 Pro72Arg polymorphism increases the susceptibility to T2DM^37^, but its mechanism in T2DM needs to be further studied. Inhibition of STAT3 can ameliorate insulin resistance, inflammation and oxidative stress in muscle and liver in T2DM model mouse^38,39^. TNF, IL6, and PTGS2 are associated with inflammation. TNF and IL6 are important inflammatory and pro-inflammatory factors. PTGS2, also known as COX-2, is a key rate-limiting enzyme in the conversion of arachidonic acid to prostaglandins. It is an important inflammatory mediator. T2DM is a low-grade inflammation state. Inflammation has been verified to be linked with the occurrence of T2DM and its complications^40,41^. Inflammation can activate mTORC1, SOCS3 and JNK. The activated mTORC1, SOCS3 or JNK can mediate the serine/threonine residues phosphorylation and the degradation of IRS1 to induce insulin resistance^42–44^. CASP3 is a key zymogen in cell apoptosis. It has been reported to be increased in glucotoxicity-induced β-cell apoptosis^45^. All these targets have verified link with the pathogenesis of T2DM.

To further explore the mechanism of JLD treatment for T2DM, GO functional and KEGG enrichment analysis were conducted. The GO functional analysis showed BP was mainly involved in gene expression, signal transduction, apoptosis and inflammation. KEGG enrichment analysis showed that PI3K-AKT signaling pathway and AGE-RAGE signaling pathway in diabetic complications were the mainly pathway related to T2DM. The activated PI3K-AKT pathway can modulate the activities of FoxO1,GSK-3β, AS160, SREBP and mTORC1 to regulate glucose, lipid and protein metabolism^34^. Abnormality of PI3K-AKT pathway causes insulin resistance (IR). Activating PI3K-AKT pathway can improve IR in T2DM model^46,47^. PI3K-AKT pathway can also inhibit streptozotocin-induced β cell apoptosis and dysfunction^48^. What’s more, PI3K-AKT pathway is also related with oxidative stress and inflammation, two important mechanisms of T2DM^49,50^. Study has verified that JLD can active AKT to reduce insulin resistance^12^. Advanced glycation end products (AGEs) refer to a group of stable end products, formed by the nonenzymatic reaction of the side-chain amino with reducing sugar. This nonenzymatic reaction is accelerated in the hyperglycemic state. AGE-RAGE interaction activate the downstream effectors, including NF-κB, JNK, MAPK, PKC and JAK/STAT. These downstream effectors can mediate insulin resistance directly by downregulating GLUT4 and insulin receptor expression, promoting serine phosphorylation of insulin receptor substrate (IRS-1), or indirectly by inducing inflammatory factors and ROS^51^.

**Figure 8:**
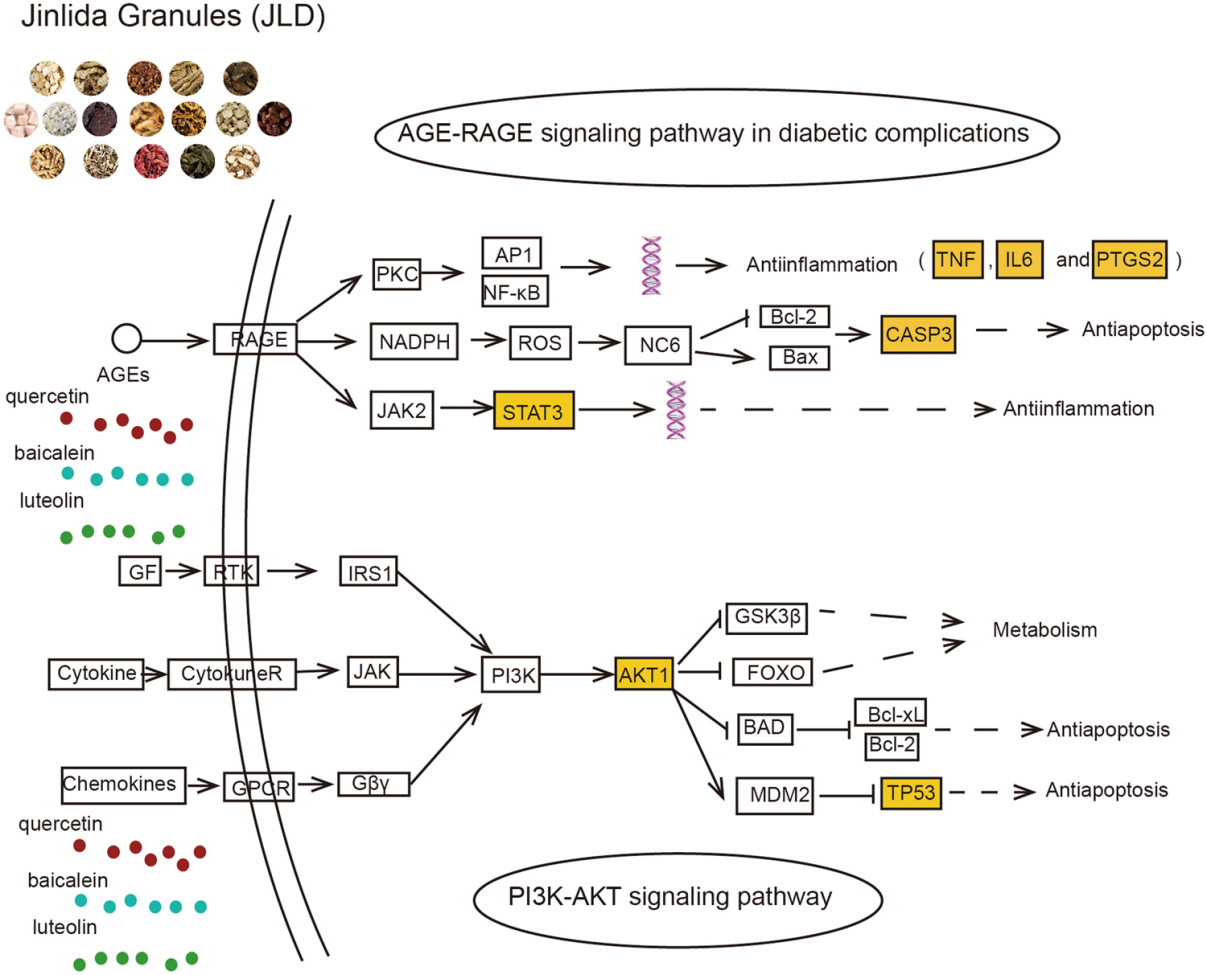
The mechanism analysis of JLD against T2DM. The highlighted nodes represent the key targets of JLD. Other nodes represent their related targets in the pathway. All these pathways were drawn according to KEGG pathway database52.

Molecular docking can predict the binding energy between target protein and active component. Molecular dynamics simulation can simulate the dynamic interaction between target protein and active component, further verify the binding stability, reveal the binding mechanism and patterns. To verify the predictions of the network pharmacology, molecular docking was first conducted to evaluate 7 key target proteins (AKT1, TNF, IL-6, TP53, PTGS2, CASP3 and STAT3) and 6 main active components (quercetin, kaempferol, luteolin, wogonin, tanshinone iia and baicalein). For each of the 7 key targets, AKT-Luteolin, TNF-Quercetin, IL-6-Quercetin, TP53-Luteolin, PTGS2- Quercetin, STAT3-Baicalein and CASP3-Baicalein had the best binding affinities. Then, molecular dynamics simulations were conducted to further verify the STAT3-Baicalein and CASP3-Baicalein interactions, which have not been performed by others. The bindings of STAT3-Baicalein and CASP3-Baicalein were stable. According to the results, quercetin, luteolin and baicalein might be the most important components. They can connect to the key targets to contribute to the therapeutic effects of JLD treatment for T2DM (Fig. 8). However, in vivo experiments were needed for further verification.

It should be noted that there were still some limitations in this study. First, the data on drugs and targets were retrieved from databases. Therefore, the data may not be comprehensive and the reliability and accuracy of the databases need to be improved. Second, network pharmacology is only an approach depending on data mining. Hence, further experimental and clinical verification are needed to be carried out.

## Conclusion

To summarize, our study demonstrated that quercetin, luteolin and baicalein may be the main active components of JLD against T2DM. AKT1, TNF, IL-6, TP53, PTGS2, CASP3 and STAT3 were the key targets. It can regulate many biological processes (gene expression, signal transduction, apoptosis and inflammation) and pathways (PI3K-AKT signaling pathway, AGE-RAGE signaling pathway in diabetic complications) to reduce insulin resistance and β cell apoptosis. This is the first time to explore the mechanism of JLD against T2DM with network pharmacology, molecular docking and molecular dynamics simulation. These findings can guide the application of JLD and further development in the treatment of JLD against T2DM.

## Methods

### Screening and collection of active components in JLD

The active components of herbs in JLD were collected from Traditional Chinese Medicine System Pharmacology Database and Analysis platform (TCMSP, http://old.tcmspw-e.com/tcmsp.php, Version: 2.3) and Bioinformatics Analysis Tool for Molecular Mechanisms of Traditional Chinese Medicine Database (BATMAN-TCM, http://bionet.ncpsb.org.cn/batman-tcm/, Version:1). Meanwhile, literatures during 2005–2024 were retrieved with “Jinlida” as the keywords for the components of JLD. Absorption, distribution, metabolism and excretion (ADME) are important indices for bioactivities of drugs. In TCMSP database, ADME-related parameters including oral bioavailability (OB) ≥ 30% and drug-likeness (DL) ≥ 0.18 were used to select active components in JLD^16^. If the components of herbs that cannot be obtained in TCMSP database, their structures were downloaded from PubChem (http://pubchem.ncbi.nlm.nih.gov, updated July 01, 2019) and were further analyzed in SwissADME database (http://swissadme.ch, updated May, 2022). Those with high gastrointestinal (GI) absorption and two or more “Yes” in DL parameters were chosen as active components^53^.

### Preparing the targets of the bioactive components

We searched for each active component related targets in TCMSP database. Other active components related targets that cannot be found in TCMSP database were searched in BATMAN-TCM database according to the criteria of score cutoff > 20, *P* < 0.05. Then using UniProt Database (http://uniprot.org), we converted all the targets into unified gene symbols, with “*Human*” organism to standardize the species.

### Screening therapeutic targets of JLD against T2DM

The key words “Type 2 diabetes”, “Type 2 diabetes mellitus” and “Diabetes, type 2” were searched in two public databases: DrugBank (https://drugbank.com, Version: 5.1.10) and Genecards Database (http://gencards.org, Version: 5.18). Then UniProt Database was utilized to standardize gene names and organism. All the genes obtained from the above two databases were summarized and duplicated genes were removed. Then T2DM related targets were mapped into the active component related targets with a Venn diagram webtool (http://www.bioinformatics.com.cn/static/others/jvenn/example.html), and the overlapped targets were regarded as the therapeutic targets of JLD against T2DM.

### Protein–protein interaction (PPI) data

The therapeutic targets of JLD against T2DM were submitted to the STRING database (http://cn.string-db.org, Version 12.0). Furthermore, the species was set to “*Homo sapiens*” and the confidence was limited to 0.04. Then a PPI data was obtained and saved as tsv file for further analysis.

### Network construction and analyses

The network was constructed as follows: (1) the JLD granule-active component network was constructed by connecting herbs of the JLD with their corresponding components; (2) the active component-disease-target network (C-D-T) was constructed by connecting the active component of JLD with the candidate therapeutic targets of JLD against T2DM; and (3) a PPI network of therapeutic targets of JLD against T2DM was built. All these networks were visualized and analyzed by Cytoscape 3.9.1 software.

To obtain core targets of JLD treatment for T2DM, CytoNCA module plug-in Cytoscape was used for topological analyses of the targets. The parameter degree centrality (DC) was employed for getting the core targets. The higher the DC value of a node, the more important the node in the network. The top 10 targets with the highest DC values were regarded as the core targets of JLD against T2DM. To pick up the hub targets, CytoHubba module plug-in Cytoscape was adopted. Two parameters including Maximum Neighborhood Component (MNC) and Maximal Clique Centrality (MCC) were employed, respectively. Meanwhile, the MCODE module was utilized for cluster analyses of the PPI network, using cut-offs: degree = 2, node score = 0.2, k-core = 2 and max depth = 100.

### Gene Ontology (GO) and Kyoto encyclopedia of gene and genomes (KEGG) pathway enrichment analyses

To further explain the role of the targets, the drug-disease intersection targets were retrieved from DAVID database (https://david.ncifcrf.gov, updated December, 2021). The species was limited to “*Homo sapiens*”. Biological process (BP), cellular component (CC) and molecular function (MF) were selected for GO analysis. GO terms and KEGG pathways analyses used *P* < 0.05 as a cutoff. The top 20 items of Go analysis and the top 30 KEGG pathways were retained. The results were visualized by using the bioinformatics online website (http://bioinformatics.com.cn/ ).

### Active component-target molecular docking

To analyze the binding affinities of component-target complexes, the molecular docking was performed with Schrodinger Maestro software 13.5. First, the crystal structure of the targets were retrieved from RCSB PDB Database (http://www.rcsb.org). The obtained crystal structures were processed with Protein Preparation Wizard module, including protein preprocessing, regenerating states of native ligand, H-bond assignment optimization, protein energy minimization and water removal. Second, the 2D structures of the active components were downloaded from the PubChem database and tranfer to 3D chiral structure with the Ligprep module. Third, the SiteMap module was used to predict the best binding site, and the Receptor Grid Generation module was used to set the most appropriate Enclosing box to perfectly wrap the predicted binding site, and on this basis, the active site of the target was obtained. Finally, the treated ligand components were docked with the active sites of the targets in the Glide docking program implementing extra-precision (XP-Glide) module and the molecular mechanics generalized Born surface area (MM/GBSA) were calculated. The results of XP-Glide docking and MM/GBSA calculation were referred to XP Gscore and MM-GBSA dG Bind, respectively. XP Gscore, less than -6, indicated the ligands had stable binding properties to the targets. MM-GBSA dG Bind, lower than − 30 kcal/mol, indicated that the binding free energy was lower and the binding was stable^54^.

### Molecular dynamics simulation

To confirm the component-target binding stability, molecular dynamics simulations were performed using the Desmond program^55^. We employed the OPLS4 force field to parameterize the target proteins and ligand components, while the SPCE model was used for the water solvent. The component-target complex was placed in a cubic water box and solved. The system was neutralized with 0.150 M chloride and sodium ions. Energy minimization was performed with the steepest descent minimization method for 50,000 steps. Subsequently, another 50,000 steps were performed to confine the positions of heavy atoms for NVT and NPT equilibration. The system temperature was maintained at 300 K, and the system pressure was maintained at 1 bar. After finishing the two equilibrations, an unrestricted simulation was carried out for 100 ns. Maestro 2023 was utilized to analyze and visualize the interactions as well as dynamic trajectory animations(Supplementary information).

### Supplementary Information

Supplementary Information.

## Data Availability

The authors declare that the data supporting the findings of this study are available within the paper and its Supplementary information files. Should any raw data files be needed in another format they are available from the corresponding author upon reasonable request.
